# Isolation and identification of bat viruses closely related to human, porcine and mink orthoreoviruses

**DOI:** 10.1099/jgv.0.000314

**Published:** 2015-12

**Authors:** Xing-Lou Yang, Bing Tan, Bo Wang, Wen Li, Ning Wang, Chu-Ming Luo, Mei-Niang Wang, Wei Zhang, Bei Li, Cheng Peng, Xing-Yi Ge, Li-Biao Zhang, Zheng-Li Shi

**Affiliations:** ^1^​ Key Laboratory of Special Pathogens and Center for Emerging Infectious Diseases, Wuhan Institute of Virology, Chinese Academy of Sciences, Wuhan, PR China; ^2^​ Guangdong Entomological Institute, Guangzhou, PR China

## Abstract

Bats have been identified as natural reservoirs of many viruses, including reoviruses. Recent studies have demonstrated the interspecies transmission of bat reoviruses to humans. In this study, we report the isolation and molecular characterization of six strains of mammalian orthoreovirus (MRV) from *Hipposideros* and *Myotis* spp. These isolates were grouped into MRV serotype 1, 2 or 3 based on the sequences of the S1 gene, which encodes the outer coat protein σ1. Importantly, we found that three of six bat MRV strains shared high similarity with MRVs isolated from diseased minks, piglets or humans based on the S1 segment, suggesting that interspecies transmission has occurred between bats and humans or animals. Phylogenetic analyses based on the 10 segments showed that the genomic segments of these bat MRVs had different evolution lineages, suggesting that these bat MRVs may have arisen through reassortment of MRVs of different origins.

Members of the viral family *Reoviridae* (respiratory enteric orphan viruses) are non-enveloped, segmented, dsRNA viruses and are classified taxonomically into two subfamilies: *Sedoreovirinae*, which contains six genera, and *Spinareovirinae*, which contains nine genera. The genus *Orthoreovirus* belongs to the subfamily *Spinareovirinae* and consists of five species: mammalian orthoreovirus (MRV), avian orthoreovirus, baboon orthoreovirus, Nelson Bay orthoreovirus and reptilian orthoreovirus. Members of the genus *Orthoreovirus* contain three large (L1–L3), three medium (M1–M3) and four small (S1–S4) genome segments (King *et al.*, 2011). Orthoreoviruses are divided into fusogenic and non-fusogenic groups, with MRV being the only member of the non-fusogenic group (Duncan, 1999). *Mammalian orthoreovirus* is the prototype species of the genus *Orthoreovirus* and is divided into four serotypes based on genetic and antigenic relatedness: type 1 Lang (T1L), type 2 Jones (T2J), type 3 Dearing (T3D) and type 4 Ndelle (T4N) (Schiff *et al.*, 2007).

Orthoreoviruses were isolated initially from the human respiratory and enteric tracts and are rarely associated with serious disease symptoms. However, some recent studies have shown that these viruses can, in some cases, cause severe disease symptoms, such as diarrhoea, pneumonia, respiratory distress syndrome and encephalitis, in humans and other animals. In Malaysia, Melaka virus, Kampar virus and Sikamat virus were isolated from adult patients with acute respiratory disease whose symptoms included high fever, myalgia, diarrhoea and severe prostration (Chua *et al.*, 2007, 2008, 2011). In Hong Kong and Japan, four individuals who were returned from Indonesia experienced high fever, myalgia and watery diarrhoea associated with orthoreovirus infections (Cheng *et al.*, 2009; Wong *et al.*, 2012; Yamanaka *et al.*, 2014). Between 2004 and 2011, orthoreoviruses were reported to cause acute meningitis in children in the USA, Canada and France, and were shown recently to cause acute gastroenteritis in an infant in Slovenia (Hermann *et al.*, 2004; Ouattara *et al.*, 2011; Steyer *et al.*, 2013; Tyler *et al.*, 2004). During the outbreak of severe acute respiratory syndrome (SARS), scientists isolated four strains of mammalian orthoreoviruses in patients with SARS and demonstrated that one of these strains could cause similar symptoms to those observed in guinea pigs and macaques with SARS (Duan *et al.*, 2003; Liang *et al.*, 2005; Song *et al.*, 2008). Orthoreoviruses have also been shown to be associated with respiratory or enteric symptoms in dogs, swine, minks and cattle (Dai *et al.*, 2012; He *et al.*, 2013; Huhtamo *et al.*, 2007; Kwon *et al.*, 2012; Lian *et al.*, 2013; Thimmasandra Narayanappa *et al.*, 2015; Zeng *et al.*, 2008; Zhang *et al.*, 2011).

In recent years, bats have been recognized as an important natural reservoir of numerous emerging viruses, such as SARS coronavirus, Ebola virus and Nipah virus (Chua *et al.*, 2002; Ge *et al.*, 2013; Leroy *et al.*, 2005). Novel orthoreoviruses have also been isolated from bats in different countries, including the first bat orthoreovirus, Nelson Bay virus, which was isolated from the fruit bat *Pteropus poliocephalus* in New South Wales, Australia (Gard & Marshall, 1973); Pulau virus, which was discovered while searching for a natural reservoir of Nipha virus on Tioman island in Malaysia (Pritchard *et al.*, 2006); and a novel orthoreovirus isolated from *Pteropus vampyrus* imported to Italy from Indonesia (Lorusso *et al.*, 2015). In addition, multiple orthoreovirus strains have been isolated from European microbats (Kohl *et al.*, 2012; Lelli *et al.*, 2013). In China, different orthoreovirus stains have been isolated from the fruit bats *Rousettus leschenaultii* and *Cynopterus sphinx* and the microbat *Rhinolophus pusillus* (Du *et al.*, 2010; Hu *et al.*, 2014; Ran *et al.*, 2007; Wang *et al.*, 2015). According to orthoreovirus classification criteria, all orthoreoviruses from fruit bats can be grouped phylogenetically into a novel proposed species, *Pteropine orthoreovirus*. In contrast, orthoreoviruses isolated from Chinese and European microbat populations belong to MRV serotypes 2 and 3, respectively.

In this study, we isolated six reoviruses from bat samples collected from seven cities or provinces throughout China from 2007 to 2012, using either Vero or *Mytotis davidii* kidney (MdKi) cells as described previously (Fig. 1, Table S1, available in the online Supplementary Material) (Li *et al.*, 2010). Five isolates were from *Hipposideros* bats (named BtMRV WIV3 to -5, -7 and -8), and one from *Myotis* bats (named BtMRV WIV2). Full-length genomic (WIV3) or nearly full-length genomic sequences (WIV2, -4 -5, -7 and -8) of these bat MRV isolates were determined using the 454/Roche sequencing platform with a genomic coverage above 300. These isolates displayed genetic divergence at the nucleotide level but shared high similarity at the amino acid level in the encoded proteins, with the exception of the S1 segments, which encoded the σ1 protein (Table 1). Based on similarities of the σ1 protein with those of prototype MRVs, BtMRV WIV2 and -8 belonged to serotype 1, BtMRV WIV3–5 to serotype 2 and BtMRV WIV7 to serotype 3 (Fig. 2). Different strains within the same serotype exhibited genetic diversity based on the different fragments. BtMRV WIV2 and -8, isolated from different bat families, shared 76–98 and 90–99 % similarity at the nucleotide and amino acid levels, respectively; their S1 segments shared 86 % similarity at the nucleotide level and 90 % similarity at the amino acid level. BtMRV WIV3 and -4 were highly similar and shared 89–98 and 95–99 % similarity at the nucleotide and amino acid levels, respectively; their S1 segments shared 93 and 95 % similarity at the nucleotide and amino acid levels, respectively. BtMRV WIV3 and -4 shared 73–99 and 79–100 % similarity with BtMRV WIV5 at the nucleotide and amino acid levels, respectively, whereas their S1 segments shared 73 % similarity with BtMRV WIV5 at the nucleotide level and 81 and 79 % similarity with BtMRV WIV5, respectively, at the amino acid level.

**Fig. 1.**
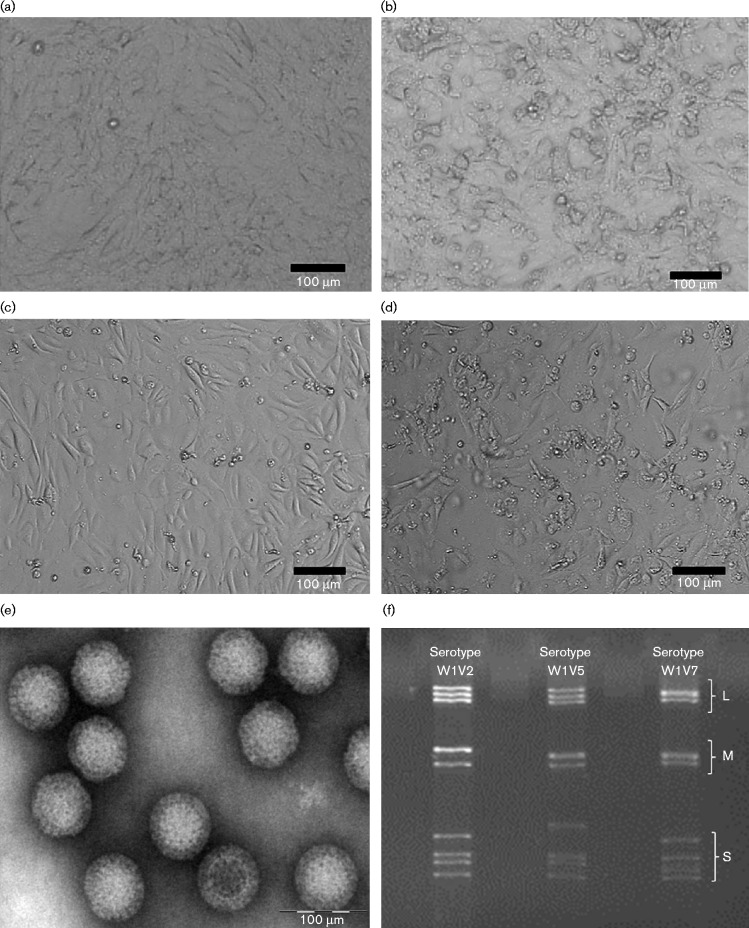
Virus culture and identification. (a, c), Uninfected Vero cells (a) and bat cells (c). (b, d), Infected Vero cells (c) and bat cells (d) showed cytopathic changes. (e) Purified viral particles imaged using electron microscopy. (f) Viral genomes observed on polyacrylamide gels. L, M and S indicate the segment group of large, medium and small sizes, respectively.

**Table 1.** Similarity of σ1 protein sequences of different bat MRV strains T1L, mammalian orthoreovirus 1 Lang; T2J, mammalian orthoreovirus 2 Jones; T3D, mammalian orthoreovirus 3 Dearing; T4N, mammalian orthoreovirus 4 Ndelle; RpMRV YN2012, mammalian orthoreovirus *Rhinolophus pusillus* YN2012; MRV2-Tou05, human mammalian orthoreovirus 2 Tou05; MRV-T3BatGe, bat orthoreovirus T3/Bat/Germany/342/08; MRV-GD1, porcine orthoreovirus strain GD-1; MRV-T3BatIt, bat orthoreovirus T3/Tadarida_teniotis/Italy/206645-51/2011; MRV1-HB-A, mink orthoreovirus.

Strain	BtMRV WIV2	BtMRV WIV3	BtMRV WIV4	BtMRV WIV5	BtMRV WIV7	BtMRV WIV8	T1L	T2J	T3D	T4N	RpMRV YN2012	MRV2-Tou05	MRV-GD1	MRV1-HB-A	MRV- T3BatGe	MRV T3BatIt
BtMRV WIV2	100	52	52	52	23	90	91	49	23	22	51	51	23	90	23	23
BtMRV WIV3	–	100	95	81	24	52	52	62	25	23	92	96	24	52	24	24
BtMRV WIV4	–	–	100	79	23	52	52	62	24	23	91	95	23	52	23	23
BtMRV WIV5	–	–	–	100	24	53	52	62	23	22	81	80	23	54	22	24
BtMRV WIV7	–	–	–	–	100	23	23	25	93	68	23	24	99	23	90	90
BtMRV WIV8	–	–	–	–	–	100	96	49	24	23	52	23	23	98	23	23

**Fig. 2.**
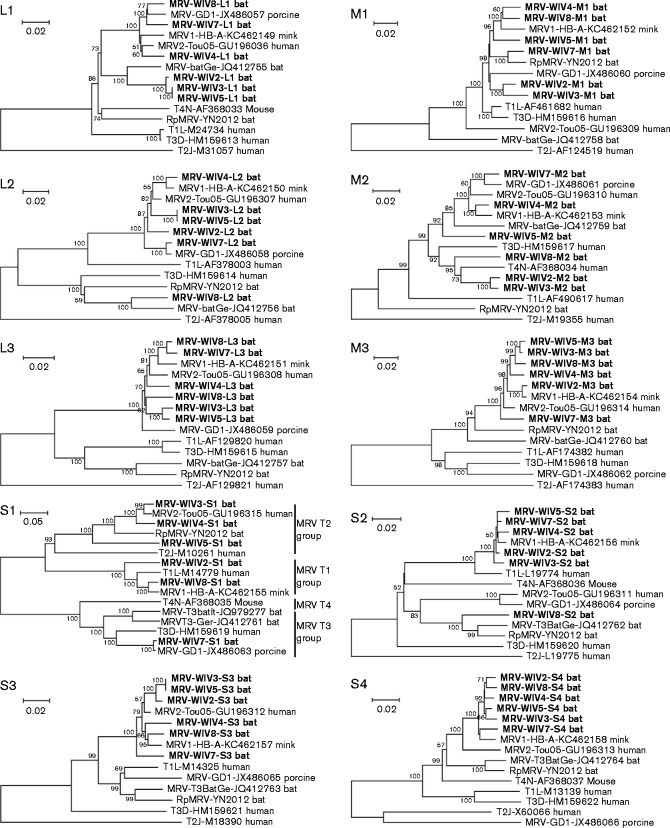
Phylogenetic analysis of bat MRVs based on the 10 segment sequences. The bat MRVs isolated in this study are shown in bold. T1L, mammalian orthoreovirus 1 Lang; T2J, mammalian orthoreovirus 2 Jones; T3D, mammalian orthoreovirus 3 Dearing; T4N, mammalian orthoreovirus 4 Ndelle; MRV1-HB-A, mink orthoreovirus HB-A; MRV-T3BatGe, bat orthoreovirus T3/Bat/Germany/342/08; MRV2-Tou05, human orthoreovirus 2 Tou05; MRV-GD1, porcine orthoreovirus strain GD-1; MRV-T3BatIt, bat orthoreovirus T3/Tadarida_teniotis/Italy/206645-51/2011; The phylogenetic tree was constructed by the neighbour-joining method using the *p*-distance model with mega6 (Tamura *et al.*, 2013) with bootstrap value of 1000. Bars, nucleotide substitutions per site. GenBank accession numbers are shown for each isolate.

One of each serotype of the bat MRVs obtained in this study was selected for further genomic characterization. The 5′ and 3′ ends of the full-length genomic segments of WIV3 were determined and shared a common terminal sequence (i.e. 5′-GCUA…UCAUC-3′), which is highly conserved in mammalian orthoreoviruses. Similar to other MRVs, the length of the 5′ UTR ranged from 12 to 33 bp, whereas that of the 3′ UTR ranged from 32 to 80 bp (Table S2). Pairwise comparisons of genomic sequences between BtMRV WIV3 and other representative MRVs showed that segment S1 had the highest nucleotide identity (96 %) with MRV2-Tou05, which was isolated from infected French children (Ouattara *et al.*, 2011), L1 had the highest identity (94 %) with the bat MRV strain MRV-T3/Bat/Germany (Kohl *et al.*, 2012) and M2 had 93 % identity with T4N, which was isolated from mice (Attoui *et al.*, 2001). The remaining seven segments were more similar to MRV1-HB-A, which was isolated from infected minks in China (Lian *et al.*, 2013) (Table S3).

Among the 10 segments of BtMRV WIV7, the L1 sequence showed the highest nucleotide identity (94 %) with MRV2-Tou05 and MRV-GD1, which was isolated from a piglet with diarrhoea in China (Dai *et al.*, 2012). The S1 sequence had the highest similarity (99 %) with MRV-GD1, and the remaining eight segments were most similar to MRV1-HB-A (Table S4).

Among the 10 segments of BtMRV WIV8, the L1 sequence showed the highest nucleotide identity (97 %) with MRV2-Tou05 and MRV-GD1; the L2 shared 93 % nucleotide identity with MRV-T3/Bat/Germany and 98 % nucleotide identity with MRV-729, which was isolated from a pig with encephalitis in Austria (GenBank accession no. JN799426); the M2 sequence showed 97 % nucleotide identity with MRV-HLJ/2007, which was also isolated from a pig in China (Zeng *et al.*, 2007); the S2 sequence had 95 % nucleotide identity with the Slovenian strain SI-MRV01, which was isolated from ill children (Steyer *et al.*, 2013), and the other segments had high similarity with MRV1-HB-A (Table S5).

Phylogenetic trees were reconstructed based on the full ORF coding sequences of bat MRVs obtained in this study and other known orthoreoviruses (Fig. 2, Tables S3–S8). The phylogenetic trees exhibited incongruent topologies based on different segments, except for the L3, M3, S3 and S4 segments, which had similar topologies and were always clustered together with MRV1-HB-A and MRV2-Tou05. BtMRV WIV2 and -8 showed different topologies in the phylogenetic trees based on the sequences of L1, L2 and M1. BtMRV WIV3, -4 and -5 showed different topologies in the phylogenetic trees based on the L1, L2, M1 and M2 segments. BtMRV WIV7, which was grouped into serotype 3, was always clustered with porcine MRVs when the tree was constructed with segments L1, L2, M2 and S1, but showed a different topology when using segments M1 and S2. These results indicated that these bat MRVs may have arisen through reassortment of MRVs of different origins.

To better understand the prevalence of MRVs in Chinese bats, 875 individual bat faecal swabs or faecal pellet samples collected in five provinces of China from 2007 to 2013 were examined by nested reverse transcription PCR. Primers targeting the sequence of all known MRV RNA-dependent RNA polymerases (RdRps) were designed and used for screening of bat MRV in our collected samples. Viral RNA was first reverse transcribed to cDNA and then used for nested PCR. The first-round primers were MRV-L1-1F (5′-TGGATATTYTCYTGGTAIATGC-3′) and MRV-L1-1R (5′-TTCACGAGCAGATAIGCCTCT-3′). The second-round primers were MRV-L1-2F (5′-TCTGGGAATGTATAGYTGGATTAG-3′) and MRV-L1-2R (5′-AGCTCCATAYTTCTCCCACTC-3′). PCR products were further confirmed by sequencing. A total of 72 (8.2 %) samples tested positive for MRV in four bat species, *Rhinolophus affinis*, *Myotis ricketti*, *Taphozous melanopogon*, and *Hipposideros pratti* (Table S9). Among these positive samples, 65 of 662 were collected monthly in one cave (Xianning, Hubei Province) in which the dominant species were *Hipposideros pratti*, *Hipposideros armiger* and *Hipposideros larvatus*. A 2-year surveillance in this cave indicated that MRVs were prevalent throughout the year, with two infection peaks observed in May and July 2011 (Table S10). We obtained a total of 76 viral partial *RdRp* gene sequences from 72 positive samples. These sequences shared 84–100 % similarity among themselves and 71–90 % identity with other known MRVs at the nucleotide level. High nucleotide similarity (99–100 %) was observed among the sequences detected in the same species, e.g. *M. ricketti* and *Rhinolophus affinis*. A neighbour-joining phylogenetic tree based on the 269 bp nucleotide sequences showed that these MRVs were placed in three different clusters (Fig. S1). The first group comprised 17 strains that were closely related to MRV2-Tou05, whereas the second group included 10 strains that were located in the same branch as MRVs detected in German bats. The remaining 44 strains formed the third cluster with human T1 and T3 MRVs.

The interspecies transmission of bat orthoreovirus from bats to humans has been documented in recent years. Melaka virus and Kampar virus, the causative agents of acute respiratory illness in humans in Malaysia, are thought to have originated from bats based on epidemiological data (Chua *et al.*, 2007, 2008). The orthoreoviruses infecting patients from Hong Kong and Japan were suspected to be of bat origin based on the phylogenetic relationships of viral sequences (Wong *et al.*, 2012). Recently, an orthoreovirus isolated from a child with acute gastroenteritis was shown to have a high similarity with a mammalian orthoreovirus found in European bats (Steyer *et al.*, 2013). Considering the diversity and wide distribution of bats and the potential transmission of viruses to humans and other animals, continued surveillance of MRVs in bats and in humans and other animals is urgently needed.

Owing to their segmented genome, reoviruses tend to be reassorted when two different strains or species infect the same host cells (Schiff *et al.*, 2007). Our phylogenetic analysis indicated that bat MRV genomic segments clearly underwent reassortment. In contrast to previous reports of MRVs isolated from bats, the viruses isolated in this study had a similar origin based on the sequences of L3, M3, S3 and S4 segments, which were closely related to human MRV2-Tou05 or mink MRV. However, the other segments had multiple origins. For example, for BtMRV WIV3, the S1 sequence was highly similar to that of human MRV, but the L1 sequence was closer to that of bat MRV isolated from European bats, the M1 sequence was closer to that of porcine MRV, the M2 sequence was closer to that of mammalian orthoreovirus 4 Ndelle, and the S2 sequence was closer to that of mink MRV. For BtMRV WIV7, the S1, L1, L2 and M2 sequences were highly similar to those of porcine MRV and may have had the same origin. In contrast, the M1 sequence was closer to that of bat MRV isolated from *Rhinolophus pusillus* in China, and the S2 sequence was closer to that of mink MRV. For BtMRV WIV8, the S1 and M1 segments were highly similar to that of mink MRV, whereas the L1 sequence was closer to that of porcine MRV. Additionally, the L2 and S2 sequences were closer to that of bat MRVs isolated from *Rhinolophus pusillus* in China and from European bats. These results suggest that the segments from different bat MRVs may have had different evolutionary lineages.

In conclusion, we found MRV serotypes 1, 2 and 3 in different bat species in China and have provided molecular evidence of interspecies transmission and multiple reassortments of these bat MRVs. These results expand our view of bats as natural reservoirs of many viruses and support the necessity of long-term surveillance of these viruses not only in animals but also in the human population.

## Supplementary Data

697 Supplementary Data
