# Supplementary material for: Isolation and identification of bat viruses closely related to human, porcine and mink orthoreoviruses
Source: J Gen Virol. 2015 Dec;96(Pt 12):3525–31. doi: 10.1099/jgv.0.000314 (PMC7081072; doi:10.1099/jgv.0.000314)
Supplement: Supplementary file 1 — Supplementary Data [file jgv-96-3525-s001.pdf]

Supplementary materials:

Supplementary Table S1: Bat samples used in virus isolation.

Supplementary Table S2: Genome characterization of BtMRV-WIV3.

Supplementary Table S3: Genomic similarity of MRV WIV3 with other MRVs.

Supplementary Table S4: Genomic similarity of MRV WIV7 with other MRVs

Supplementary Table S5: Genomic similarity of MRV WIV8 with other MRVs

Supplementary Table S6: Genomic similarity of MRV WIV2 with other MRVs.

Supplementary Table S7: Genomic similarity of MRV WIV4 with other MRVs.

Supplementary Table S8: Genomic similarity of MRV WIV5 with other MRVs.

Supplementary Table S9: Prevalence of MRV in Chinese bats

Supplementary Table S10: Prevalence of MRV in bat colony in Xianning, Hubei Province

Supplementary Figure S1. Phylogenetic analysis of bat MRVs detected in Chinese bats based on partial RdRp sequences. The sequences identified in this study are shown in bold, and numbers of positive samples are indicated in parentheses. T1L, mammalian orthoreovirus 1 Lang; T2J, mammalian orthoreovirus 2 Jones; T3D, mammalian orthoreovirus 3 Dearing; T4N, mammalian orthoreovirus 4 Ndelle; MRV-T3BatGe, bat orthoreovirus T3/Bat/Germany/342/08; MRV2-Tou05, human orthoreovirus 2 Tou05; NBV, Nelson Bay orthoreovirus. XN, Xianning city; YN, Yunnan Province; GX, Guangxi Province. NJ tree, the number of bootstrap replicates is 1000.

Supplementary Table S1: Bat samples used in virus isolation

Sample	Location (Province)	Bat species	Cell infected	Virus isolates
2007TJF	Tianjin	*Myotis ricketti*	MdKi*	BtMRV WIV2
2009YNF	Yunnan	*Myotis* sp.		--
2001104XNF	Xianning, Hubei	*Hipposideros pratti*; *Hipposideros armiger;* *Hipposideros larvatus*	Vero	--
2001105XNF/U				--
2001106XNF/U				--
2001107XNU				BtMRV WIV3
2001108XNF/U				BtMRV WIV4
2001109XNF/U				BtMRV WIV5
20011CQF/U	Chongqing	*Hipposideros pratti*		BtMRV WIV7
20011SCF/U	Sichuan	*Hipposideros pratti;* *Hipposideros larvatus*		--
2011GXF	Guangxi	*Taphozous melanopogon*		--
XN2811	Xianning, Hubei	*Hipposideros pratti*		BtMRV WIV8

*, Myotis Kidney cell line, previously called BK cell line; U, urine; F, feces.

Supplementary Table S2: Genome characterization of BtMRV WIV3

Segment	Size (bp)	5' End	3' End	5' UTR	3' UTR	Encoding protein	Protein size (aa)	Protein function
L1	3854	5'GCUA	UCAUC3'	18bp	32bp	λ3	1267	RNA-dependent RNA polymerase
L2	3915			12bp	33bp	λ2	1289	Guanyltransferase, methyltransferase
L3	3901			13bp	60bp	λ1	1275	Helicase, binds dsRNA, NTPase
M1	2304			13bp	80bp	μ2	736	NTPase
M2	2203			29bp	47bp	μ1	708	Cell penetration, apoptosis
M3	2241			18bp	57bp	μNS	721	Nucleates viral inclusion bodies
S1	1437			13bp	38bp	σ1, σ1s	461, 114	Viral attachment
S2	1331			18bp	56bp	σ2	418	Inner capsid structural protein
S3	1198			27bp	70bp	σNS	366	ssRNA-binding
S4	1196			32bp	66bp	σ3	365	dsRNA-binding, modulation of, cellular interferon response

Supplementary Table S3: Genomic similarity of MRV WIV3 with other MRVs.

		Similarity (%) with different MRV serotype strains (nucleotide/amino acid)								
MRV WIV3	Protein (aa)	T1L	T2J	T3D	T4N	MRV-T3BatGe	MRV2-Tou05	MRV1-HB-A	MRV-GD1	RpMRV YN2012
L1(λ3)	1267	89/98	75/92	89/98	89/98	94/98	90/99	90/98	89/98	89/99
L2(λ2)	1289	86/96	73/87	76/93	NA*	76/92	97/99	96/99	95/98	76/92
L3(λ1)	1275	84/98	77/96	84/98	NA	86/98	97/99	96/99	96/99	84/98
M1(μ2)	736	94/98	70/80	94/97	NA	86/95	89/96	93/96	93/96	93/97
M2(μ1)	708	85/98	76/97	91/98	93/99	89/98	89/99	89/99	89/98	83/97
M3(μNS)	721	85/96	70/82	85/96	NA	90/98	97/99	97/99	84/95	91/98
S1(σ1)	461	57/52	62/62	40/24	39/23	39/23	96/96	57/52	41/24	85/92
S2(σ2)	418	95/99	77/94	84/98	85/97	85/98	84/98	98/98	84/97	86/98
S3(σNS)	366	90/98	74/86	84/97	NA	89/98	97/99	96/98	88/98	89/99
S4(σ3)	365	87/97	79/92	86/97	90/94	91/98	94/99	97/99	76/86	91/98

T1L, mammalian orthoreovirus 1 Lang; T2J, mammalian orthoreovirus 2 Jones; T3D, mammalian orthoreovirus 3 Dearing; T4N, mammalian orthoreovirus 4 Ndelle; MRV-T3BatGe, bat orthoreovirus T3/Bat/Germany/342/08; MRV2-Tou05, human orthoreovirus 2 Tou05; MRV1-HB-A, mink orthoreovirus isolate HB-A; RpMRV YN2012, mammalian orthoreovirus *Rhinolophus pusillus* YN2012; MRV-GD1, porcine orthoreovirus strain GD-1; L, large segment; M, medium segment; S, small segment. *NA, not available.

Supplementary Table S4: Genomic similarity of MRV WIV7 with other MRVs

MRV WIV7	Protein (aa)	Similarity (%) with different MRV serotype strains (nucleotide/amino acid)								
		T1L	T2J	T3D	T4N	MRV-T3BatGe	MRV2-Tou05	MRV1-HB-A	MRV-GD1	RpMRV YN2012
L1(λ3)	1267	89/97	75/91	89/98	89/97	90/98	97/99	96/98	97/98	90/98
L2(λ2)	1289	86/96	72/87	77/93	NA*	76/92	96/99	95/98	99/99	76/93
L3(λ1)	1275	84/98	77/95	84/98	NA	83/98	96/99	97/99	95/99	84/98
M1(μ2)	736	94/98	70/80	94/97	NA	86/95	89/96	94/97	93/97	97/99
M2(μ1)	708	85/98	76/97	89/98	88/98	92/99	96/99	96/99	97/99	84/97
M3(μNS)	721	85/96	70/82	85/96	NA	90/98	97/98	95/98	83/95	91/97
S1(σ1)	455	39/23	40/25	85/93	67/68	80/90	40/23	39/23	99/99	39/23
S2(σ2)	418	96/99	77/94	84/98	85/97	85/98	84/98	99/99	84/97	86/99
S3(σNS)	366	89/97	74/86	84/96	NA	89/97	97/98	95/98	87/97	88/98
S4(σ3)	365	87/96	78/91	86/96	90/93	91/97	94/98	97/98	75/85	91/97

L, large segment; M, medium segment; S, small segment. *NA, not available.

Supplementary Table S5: Genomic similarity of MRV WIV8 with other MRVs

MRV WIV8	Protein (aa)	Similarity (%) with different MRV serotype strains (nucleotide/amino acid)								
		T1L	T2J	T3D	T4N	MRV-T3BatGe	MRV2-Tou05	MRV1-HB-A	MRV-GD1	RpMRV YN2012
L1(λ3)	1267	89/97	75/92	89/98	89/97	90/98	97/99	96/99	97/99	90/98
L2(λ2)	1289	75/92	72/86	87/97	NA*	93/98	76/93	76/93	76/93	86/97
L3(λ1)	1275	83/98	77/95	84/98	NA	84/98	96/99	95/99	95/99	84/98
M1(μ2)	736	95/98	70/80	94/97	NA	86/96	90/96	96/97	93/96	94/97
M2(μ1)	708	85/98	76/97	90/98	93/99	89/98	89/99	89/98	89/98	83/97
M3(μNS)	721	84/96	70/82	85/95	NA	90/98	97/98	96/99	84/94	91/98
S1(σ1)	470	91/96	55/49	39/24	36/23	38/23	57/51	96/98	39/23	58/52
S2(σ2)	418	86/98	76/93	83/98	84/96	93/98	86/98	85/98	86/97	93/99
S3(σNS)	366	90/97	73/86	85/97	NA	89/97	98/98	97/99	88/98	88/98
S4(σ3)	365	86/96	78/91	86/96	90/94	91/98	94/98	97/99	76/85	92/98

L, large segment; M, medium segment; S, small segment. *NA, not available.

Supplementary Table S6: Genomic similarity of MRV WIV2 with other MRVs.

MRV WIV2	Protein (aa)	Similarity (%) with different MRV serotype strains (Nucleotide / amino acid)								
		T1L	T2J	T3D	T4N	MRV-T3BatGe	MRV2-Tou05	MRV1-HB-A	MRV-GD1	RpMRV YN2012
L1(λ3)	1267	89/98	75/92	89/98	89/98	94/99	90/99	90/98	90/98	90/99
L2(λ2)	1289	86/97	72/87	77/93	NA	75/92	97/99	96/98	96/98	76/93
L3(λ1)	1275	84/98	77/95	84/98	NA	84/98	97/99	97/99	95/99	84/98
M1(μ2)	736	95/98	69/80	95/98	NA	86/96	90/96	94/97	94/97	94/98
M2(μ1)	708	85/98	76/97	91/98	93/99	89/98	89/99	88/98	88/98	83/97
M3(μNS)	721	85/95	70/82	84/95	NA	90/97	97/98	99/99	84/95	91/98
S1(σ1)	470	87/91	54/49	38/23	36/22	38/23	57/51	85/90	38/23	57/51
S2(σ2)	418	96/99	77/94	84/98	85/97	85/98	84/98	99/99	84/97	86/99
S3(σNS)	366	90/97	74/86	85/97	NA	90/98	98/99	96/98	88/98	89/98
S4(σ3)	365	87/96	78/91	86/96	90/93	91/98	94/98	97/98	76/85	91/98

L, large segment; M, medium segment; S, small segment. *NA, not available.

Supplementary Table S7: Genomic similarity of MRV WIV4 with other MRVs.

		Similarity (%) with different MRV serotype strains (Nucleotide / amino acid)								
MRV WIV4	Protein (aa)	T1L	T2J	T3D	T4N	MRV-T3BatGe	MRV2-Tou05	MRV1-HB-A	MRV-GD1	RpMRV YN2012
L1(λ3)	1267	89/97	75/92	89/98	89/97	91/98	97/99	96/99	90/98	90/98
L2(λ2)	1289	86/98	72/87	76/93	NA	75/93	97/99	96/99	95/98	76/93
L3(λ1)	1275	84/98	76/95	84/98	NA	84/98	96/99	95/99	95/99	84/98
M1(μ2)	736	95/98	70/80	94/97	NA	86/95	90/96	96/97	93/96	94/97
M2(μ1)	708	85/98	76/97	89/98	89/98	93/99	96/100	97/99	96/99	84/98
M3(μNS)	721	85/95	70/82	84/95	NA	90/98	97/98	97/99	84/95	91/98
S1(σ1)	461	57/52	62/62	40/24	39/23	38/23	93/95	56/52	40/23	85/91
S2(σ2)	418	95/99	76/94	84/99	85/97	85/99	84/98	98/99	83/98	85/99
S3(σNS)	366	89/96	73/85	84/96	NA	89/97	97/98	96/99	87/97	89/97
S4(σ3)	365	87/96	78/91	87/96	90/93	91/98	94/98	97/98	75/85	92/98

L, large segment; M, medium segment; S, small segment. *NA, not available.

Supplementary Table S8: Genomic similarity of MRV WIV5 with other MRVs.

MRV WIV5	Protein (aa)	Similarity (%) with different MRV serotype strains (Nucleotide / amino acid)								
		T1L	T2J	T3D	T4N	MRV-T3BatGe	MRV2-Tou05	MRV1-HB-A	MRV-GD1	RpMRV YN2012
L1(λ3)	1267	89/98	75/92	89/98	89/98	94/98	90/99	90/98	89/98	89/99
L2(λ2)	1289	85/96	72/87	76/93	NA	75/92	97/99	96/99	95/98	76/92
L3(λ1)	1275	84/98	77/96	84/98	NA	84/98	96/99	96/99	95/99	84/98
M1(μ2)	736	95/98	70/80	94/97	NA	86/95	90/96	95/96	93/96	94/97
M2(μ1)	708	85/98	76/97	90/99	91/99	91/99	93/99	93/99	92/99	83/97
M3(μNS)	721	84/95	70/82	85/96	NA	90/98	97/98	96/98	83/94	91/98
S1(σ1)	461	56/52	60/62	39/23	38/22	39/22	76/80	57/54	39/23	75/81
S2(σ2)	418	95/99	77/94	84/98	85/97	85/98	84/98	98/99	84/97	85/99
S3(σNS)	366	90/98	74/86	84/97	NA	89/98	97/99	96/98	88/98	89/99
S4(σ3)	365	87/96	78/91	86/96	90/94	91/98	94/98	97/99	76/86	92/98

L, large segment; M, medium segment; S, small segment. *NA, not available.

Supplementary Table S9: Prevalence of MRV in Chinese bats

Bat species	Location	Date	Sample	Number	Positive
Rhinolophus affinis	Yunnan	2013.04	Fecal swab	46	3(6.5)
Hipposideros pratti	Sichuan	2011.09	Fecal pellet	48	0(0)
Hipposideros sp.	Henan	2013.05	Fecal pellet	54	0(0)
Myotis ricketti	Tianjin	2007.07	Fecal swab	34	3(8.8)
Taphozous melanopogon	Guangxi	2011.07	Fecal pellet	31	1(3.2)
Hipposideros pratti; Hipposideros armiger; Hipposideros larvatus	Xianning,Hubei	2011.04-2012.09	Fecal pellet	662	67(10.1)
	Total			875	74(8.5)

Supplementary Table S10: Prevalence of MRV in bat colony in Xianning, Hubei Province

Sampling date	Sample	Positive sample (%)
20110411	35	1(2.86)
20110513	48	20(41.67)
20110604	48	5(10.42)
20110705	49	24(48.98)
20110821	48	3(6.25)
20110929	56	5(8.93)
20120507	53	1(1.89)
20120602	48	3(6.25)
20120619	47	0(0.00)
20120704	48	2(4.17)
20120720	48	1(2.08)
20120816	48	1(2.08)
20120906	41	0(0.00)
20120927	45	1(2.22)
Total	662	67(10.12)

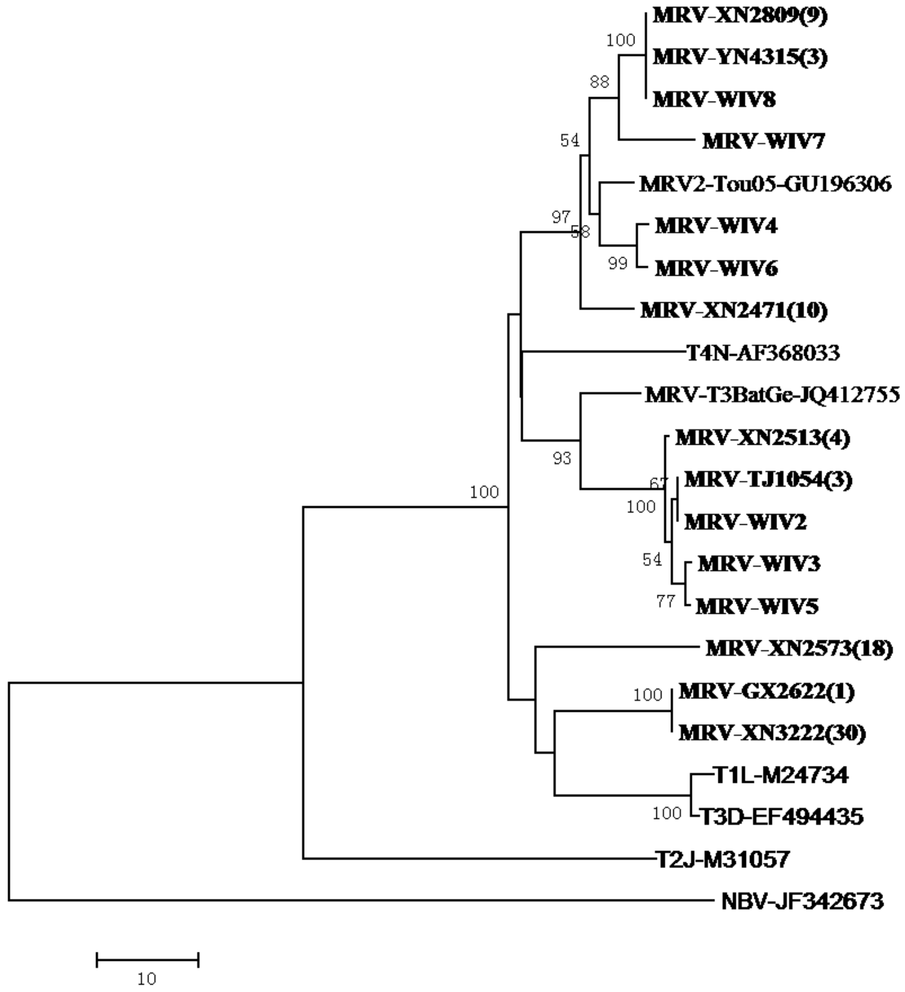

Supplementary Figure S1
